# Biological activities and chemical compositions of slime tracks and crude exopolysaccharides isolated from plasmodia of *Physarum polycephalum* and *Physarella oblonga*

**DOI:** 10.1186/s12896-017-0398-6

**Published:** 2017-11-09

**Authors:** Tuyen T.M Huynh, Trung V. Phung, Steven L. Stephenson, Hanh T.M Tran

**Affiliations:** 1 School of Biotechnology, International University, VNU-HCM, Block 6, LinhTrung Ward, Thu Duc District, Ho Chi Minh City, 70000 Vietnam; 2 Institute of Chemical Technology, Vietnam Academy of Science and Technology, 01-Mac Dinh Chi Street, District 1, Ho Chi Minh City, 70000 Vietnam; 3 Department of Biological Sciences, University of Arkansas, Fayetteville, AR 72701 USA

**Keywords:** *Candida albicans*, HepG2, Mcf-7, Monomer composition, Slime molds

## Abstract

**Background:**

The myxomycetes derive their common name (slime molds) from the multinucleate trophic stage (plasmodium) in the life cycle, which typically produces a noticeable amount of slimy materials, some of which is normally left behind as a “slime track” as the plasmodium migrates over the surface of a particular substrate. The study reported herein apparently represents the first attempt to investigate the chemical composition and biological activities of slime tracks and the exopolysaccharides (EPS) which cover the surface of the plasmodia of *Physarum polycephalum* and *Physarella oblonga*.

**Results:**

Chemical analyses indicated that the slime tracks and samples of the EPS consist largely of carbohydrates, proteins and various sulphate groups. Galactose, glucose and rhamnose are the monomers of the cabohydrates present. The slime tracks of both species and the EPS of *Phy. oblonga* contained rhamnose, but the EPS of *Ph. polycephalum* had glucose as the major monomer. In term of biological activities, the slime tracks displayed no antimicrobial activity, low anticancer activity and only moderate antioxidant activity. However, EPSs from both species showed remarkable antimicrobial activities, especially toward *Candida albicans* (zone of inhibition ≥20 mm). Minimum inhibitory concentrations of this fungus were found to be 2560 μg/mL and 1280 μg/mL for EPS from *Phy. oblonga* and *Ph. polycephalum*, respectively. These EPS samples also showed moderate antioxidant activities. However, they both displayed cytotoxicity towards MCF-7 and HepG2 cancer cells. Notably, EPS isolated from the plasmodium of *Phy. oblonga* inhibited the cell growth of MCF-7 and HepG2 at the half inhibitory concentration (IC50) of 1.22 and 1.11 mg/mL, respectively.

**Conclusions:**

EPS from *Ph. polycephalum* plasmodium could be a potential source of antifungal compounds, and EPS from *Phy. oblonga* could be a potential source of anticancer compounds.

## Background

Exopolysaccharides (EPSs) are macromolecules mainly composed of carbohydrate residues, which are secreted by microorganisms into the surrounding environment. EPSs can serve as centers for bacterial cell aggregation, as nutrient sources and also form a protective barrier for the cell against harsh external conditions [1]. Microbial EPSs have gained a great deal of interest due to their potential biological activities [2]. EPSs isolated from bacteria and fungi have been found to possess inhibitory activities against gram positive and negative bacteria and the H1N1 virus [3–5]. EPSs isolated from bacteria and fungi have a significant scavenging ability against superoxide, hydroxyl and DPPH radicals [6–8]. Microbial EPSs also represent a promising source of anticaner agents. Cell-bound galactan exoplysaccharide of *Lactobacillus plantarum*, at a concentration of 600 μg/mL, showed cytotoxic effects of about 56.34% against the human liver carcinoma (HepG2) cell line [9]. Osama et al. [5] found that EPS isolated from *Bacillus marinus* showed a strong antitumor property against breast cancer (MCF-7) cell lines and alveolar basal epithelia (A-549) cell lines at concentration of 100 μg/mL. In addition, EPS from *Aspergillus aculeatus* displayed a strong anti-proliferation effect on human cervical carcinoma cells (Hela), human breast carcinoma cells (MCF-7) and gastric carcinoma cells (MGC-803) with inhibition rates of 53.9%, 29.1% and 34.1%, respectively, at a concentration of 1000 μg/mL for 48 h [10].

The myxomycetes are a group of primitive phagotrophic eukaryotes. The myxomycete life cycle consists of two very different trophic stages—uninucleate amoebae and a distinctive multinucleate structure, the plasmodium. Under favorable conditions, the plasmodium converts into fruiting bodies [11]. Having the characteristics of both fungi and protozoans make the myxomycetes an unusual group of microorganisms. More than 100 secondary metabolites have been isolated from myxomycetes, and many of among those are novel bioactive compounds [12]. In addition to potential antimicrobial compounds such as a new glycerolipid (bahiensol) isolated from the plasmodium of *Didymium bahiense* [13], stigmasterol and fatty acids obtained from plasmodial extracts of *Phy. oblonga* [14], some remarkable anticancer compounds from myxomycetes have also been reported. Cyclic phosphatidic acid (CPA), a novel bioactive lipid isolated from *Ph. polycephalum* was found to have ability to inhibit cancer cell invasion and metastasis [15]. In addition, two new bisindole alkaloids isolated from the fruiting bodies of *Lycogala epidendrum* showed cytotoxicity against HeLa cells and Jurkat cells with relatively low IC50 values [16]. In similar research, Kehokorins A, a novel dibenzofuran isolated from the fruiting bodies of *Trichia favoginea var. persimilis* was found to have significantly high cytotoxicity toward HeLa cells with an IC50 value of 1.5 μg/mL [17].

Among the myxomycetes, those members of the Physarales (e.g., *Physarum polycephalum*) often form large plasmodia and are relatively easy to culture on synthetic media. When cultured in liquid media, microplasmodia are formed instead of plasmodia. Both microplasmodia and plasmodium lack cell walls. On solid media, the plasmodium is a slimy mass of protolasm which is capable of moving around. In the absence of cell walls, the slime sheath represents the only protection from injury and the environment, and material from the slime sheath is left behind as a slime track as the plasmodium migrates over the surface of a given substrate [18]. There have been a few studies of the chemical composition of EPSs isolated from microplasmodia in liquid culture, but there appear to be no studies of the properties of EPS and slime tracks isolated from solid cultures of myxomycete plasmodia. The chemical characteristics of the EPSs seem to strongly depend upon the culture media used. McCormick et al. [19] found that *Ph. polycephalum* microplasmodial cultures started to produce more EPS when the cells were converted into spherules and reported that the EPS is a sulfated galactose polymer containing trace amounts of rhamnose. Simon and Henney [20] reported that the EPS was a glycoprotein. More recently, Sperl [21] found the EPSs produced by *Ph. polycephalum* consisted of two galactans with different ratios of phosphorous and sulfur. To the best of our knowledge, there has been only one report on the biological activity of myxomycete EPS, and this was published by Asgari and Henney [22]. Their research found that the EPS secrected by the microplasmodia of *Physarum flavicomum* in liquid culture was composed mainly of glycoprotein and could inhibit the cell growth and division of *Bacillus subtilis*.

Given the fact that microbial EPSs have been found to have potential biological activities and myxomycete plasmodia produce a noticeable amount of slimy materials, it seemed worthwhile to evaluate the biological activities (antimicrobial, antioxidant and anticancer activities) and to determine the chemical characteristics of slime tracks and EPS samples isolated from *Physarella oblonga* and *Physarum polycephalum*. These two species were chosen because of their sample availability and their ease to culture.

## Results and discussion

### EPS production of *Phy. oblonga* and *Ph. polycephalum*

**Fig. 1:**
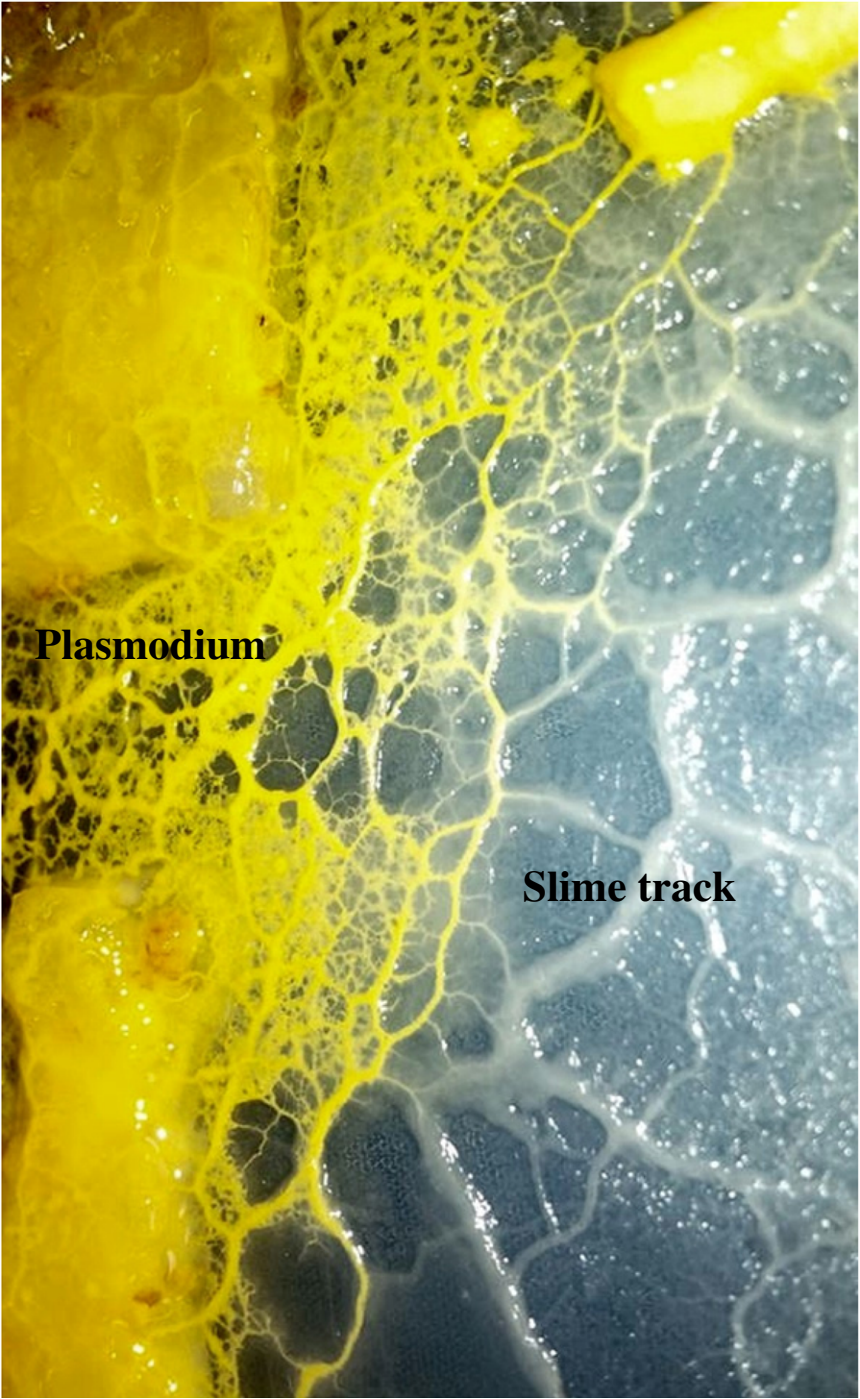
Plasmodium and slime track

The medium used for cultivation of myxomycete plasmodia was adapted from the research of Henney and Henney [23]. We attempted to replace glucose in the original medium with other carbon sources (e.g., oyster mushroom powder [since the oyster mushroom is one of the favorite food sources of some myxomycete plasmodia in the nature], rice bran and galactose). However, preliminary results showed that *Ph. polycephalum* preferred glucose and *Phy. oblonga* grew better in water agar without glucose (*Phy. oblonga* has agar hydrolytic activity). As such, for slime track and EPS production, typical plasmodia of *Ph. polycephalum* and *Phy. oblonga* were transferred to nutrient and water agar, respectively, and incubated under dark condition at 25 °C for 7 days (Fig 1). The amounts of slime track material and EPSs obtained are presented in Table 1. The amounts of slime track material obtained from both species were higher than those of EPSs still in contact with the plasmodium.

**Table 1:** Amounts of slime track and EPSs isolated from cultures of *Phy. oblonga* and *Ph. polycephalum*

	Sample	Amount (g/L)
*Phy. oblonga*	Slime track	0.51 ± 0.12
	EPS	0.28 ± 0.09
*P. polycephalum*	Slime track	0.65 ± 0.10
	EPS	0.43 ± 0.07

### Chemical composition of the slime track and EPS samples from *Phy. oblonga* and *Ph. polycephalum*

The carbohydrate, protein and sulfate contents of EPSs are listed in Table 2. The total carbohydrate content of the samples varied from 55 to 82% according to the phenol-sulfuric acid method. Sulfated groups and protein made up small proportions (Table 2). In general, the EPS and slime track of *Phy. oblonga* had greater amounts of carbohydrate compared to those of *Ph. polycephalum*. However, the samples from the latter species had higher percentages of sulfate content. When comparisons are made between the slime track and EPS samples of each species, the amounts of carbohydrates of the slime tracks were higher than that of the EPS, and this applied for both species.

**Table 2:** Total carbohydrate, protein and sulfate contents of the slime track and EPSs

Crude EPS	Total carbohydrate (%)	Protein (%)	Sulfate content (%)
*Phy. oblonga* slime track	82.13 ± 0.037	7.58 ± 0.02	1.50 ± 0.37
*Phy. oblonga* EPS	76.80 ± 0.052	19.81 ± 0.02	2.44 ± 0.05
*Ph. polycephalum* slime track	63.94 ± 0.056	12.70 ± 0.05	5.23 ± 0.04
*Ph. polycephalum* EPS	56.42 ± 0.061	30.94 ± 0.04	11.26 ± 0.02

**Fig. 2:**
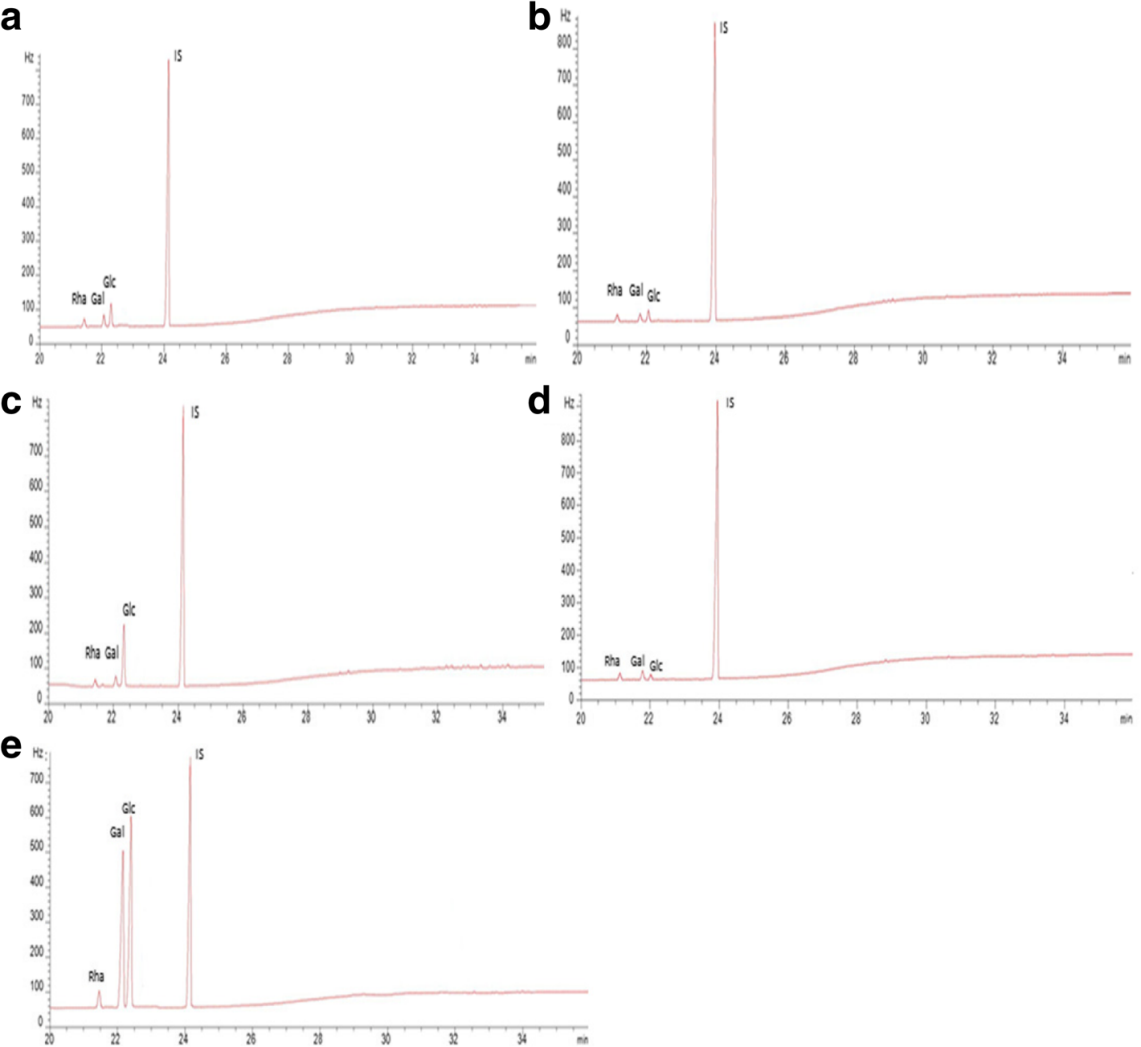
Chromatograms of GC analysis of the monosaccharide composition of slime tracks and EPSs. The chromatogram of *Phy. oblonga* EPS (**a**)*, Phy. oblonga* slime trạck (**b**), *Ph. polycephalum* EPS (**c**), *Ph. polycephalum* slime track (**d**) and standard sugars (**e**) was developed using values of GC. Galactose (Gal), glucose (Glc), rhamnose (Rha) were used as standard sugars. Inositol (IS) was used as internal reference

The slime tracks and EPSs were depolymerized by using the TFA hydrolysis method. The monosaccharide compositions of the EPSs produced by *Phy. oblonga* and *P. polycephalum* were detected by TLC, and their quantities were measured by GC-FID analysis. The data obtained data are displayed in Fig. 2 and Table 3.

**Table 3:** Monomer compositions of crude EPSs obtained from cultures of *Ph. polycephalum* and *Phy. oblonga*

Monomer composition (%*w*/w)	POS	POP	PPS	PPP
Galactose	15.65	14.09	19.43	9.47
Glucose	17.96	23.32	9.1	50.87
Rhamnose	66.37	62.58	71.46	39.65

POS and POP are slime track and EPS samples from *Phy. oblonga*, whereas PPS and PPP are slime track and EPS samples from *Ph. polycephalum*

Table 3 showed that the slime track and EPS samples contained glucose, galactose and rhamnose and rhamnose was the major monosaccharide of the EPS from *Phy. oblonga* and the slime tracks of both species**,** for which it accounted for 66.37%, 62.58% and 71.46%, respectively. In contrast, EPS from *Ph. polycephalum* was composed mainly of glucose (50.87%).

The present study is the first to determine the monomer compositions of EPSs isolated from *Phy. oblonga*. However, with *Ph. polycephalum*, the results reported have varied from one study to another. Extracellular slime from broth cultures (containing glucose as the carbon source) of *Ph. polycephalum* was found to contain galactose, sulfate, and trace amounts of rhamnose [19]. However, Simon and Henny [20] found that slime production of *Ph. rigidum*, *Ph. flavicomun* and *Ph. polycephalum* contained a single sugar component of galactose when cultured on media containing glucose as the carbon source. Similar results for *Ph. polycephalum* were also reported by Farr et al. [24]. In general, monomer compositions and their ratios in microbial EPSs are influenced by the carbon source in the culture medium [25, 26]. However, with the myxomycetes, there would appear to be some other factors involved. *Ph. polycephalum* in our study was cultured on a glucose-based solid medium, but the monomer composition was completely different from what has been reported in other studies. It is possible that plasmodia produce different kinds of slime material as compared to microplasmodia.

### Antimicrobial activity of EPSs against pathogens

Antimicrobial activities of the EPS and slime track samples as determined by the agar diffusion method are presented in Table 4.

**Table 4:** Antimicrobial activities of EPS and slime track samples from *Phy. oblonga* and *Ph. polycephalum*

Microorganism	Inhibition zone (mm)				
	Antibiotics	POS	POP	PPS	PPP
*B. cereus*	19.75 ± 0.23	–	–	–	–
*S. aureus*	23.00 ± 0.41	–	12.75 ± 0.28	–	10.05 ± 0.40
*E. coli*	18.50 ± 0.35	–	–	–	–
*S. typhi*	16.78 ± 0.52	–	–	–	8.30 ± 0.12
*C. albicans*	32.00 ± 0.22	–	20.05 ± 0.36	–	23.00 ± 0.28

*D* diameter of inhibition zone; −: No inhibition observed (D < 10 mm); +: 10 < D < 15 mm; ++: 15 < D < 20 mm; +++: D > 20 mm

POS and POP are slime track and EPS samples from *Phy. oblonga*, whereas PPS and PPP are slime track and EPS samples from *Ph. polycephalum*

The results indicate that there were significant differences in antimicrobial activities among the samples. The slime tracks of both two species did not exhibit any inhibitory activity against the strains of microbes tested. This could be explained by the theory that myxomycete plasmodia leave slime tracks behind when migrating simply to mark the area which has been exploited for food resources [12]. In contrast, isolated EPS from plasmodia showed promising activities towards *S. aureus* and *C. albicans*, whereas *C. albicans* was found to be the most susceptible to the EPSs from both species (zone of inhibition ≥20 mm) (Table 4). The antimicrobial activities of the EPSs which are still in contact with the plasmodia would be explained by the possibility that these compounds would protect the plasmodia from external factors, including other microorganisms.

The results obtained for antimicrobial activities in the present study agree with those reported in some previous studies relating to the antimicrobial property of microbial EPSs. Asgari and Henney [22] found that the cell growth and division of *Bacillus subtilis* (a gram positive bacterium) was inhibited by slime secreted by *Ph. flavicomun*. The degradation of the cell wall caused morphological changes such as swollen cells or cell lysis. Li et al. [27] found that EPS from *Lactobacillus plantarum* exhibited inhibitory activities against *S. aureus* and *C. albicans*. EPS from *Enterobacter faecalis* showed significantly high activity toward *C. albicans* [28].

The MIC values of the EPS samples from *Ph. polycephalum* and *Phy. oblonga* were studied against *C. albicans* and *S. aureus*. The data obtained are shown in Table 5.

**Table 5:** Minimum inhibitory concentration (MIC) and minimum bactericidal concentration (MBC) or minimum fungicidal concentration (MFC) of EPS and slime track samples from *Phy. oblonga* and *Ph. polycephalum*

	MIC (MBC or MFC) μg/mL		
Microorganism	POP	PPP	Standard antibiotic^a^
*S. aureus*	5120 (5120)	5120 (20480)	1280
*C. albicans*	2560 (10240)	1280 (5120)	640

^a^Standard antibiotics include gentamycin (antibacterial drug) and ketoconazole (antifungal drug). POP indicates EPS samples from *Phy. oblonga*, whereas PPP are EPS samples from *Ph. polycephalum*

With respect to their ability against *S. aureus*, the MIC value of the *Ph. polycephalum* EPS was almost the same with that of *Phy. oblonga*. However, EPS from *Ph. polycephalum* showed much better antifungal activity, since the MIC value (1280 μg/mL) of the EPS from this species against *C. albicans* was just about a half that from *Phy. oblonga* (2560 μg/mL) and twice when compared with the standard antifungal drug (640 μg/mL) (Table 5). However, this EPS is not yet purified. Nevertheless, EPS from *Ph. polycephalum* appears to have the potential for treatment of *C. albicans*. However, it should be noted that the MBC or MFC values are higher than the MIC values. This suggests that the compound would easily inhibit microbial growth at low concentrations, but leading to actual microbial death would require higher doses.

### Antioxidant activity

**Fig. 3:**
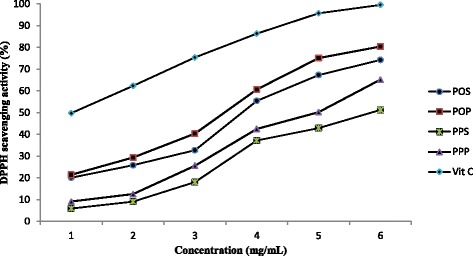
Antioxidant activities of EPSs of *Phy. oblonga* and *Ph. polycephalum* in vitro*.* POS and POP are slime track and EPS samples from *Phy. oblonga*, whereas PPS and PPP are slime track and EPS samples from *Ph. polycephalum*

In this part of our study, in vitro antioxidant activities of the EPS samples with the concentration range of 0–6.0 mg/mL from *Phy. oblonga* and *Ph. polycephalum* were determined by DPPH assay and compared with that of ascorbic acid. Figure 3 illustrates that there was not a major difference observed between radical scavenging ability of slime track and EPS extracts from *Phy. oblonga* and *Ph. polycephalum* at an initial concentration 1.0 mg/mL. However, at the higher sample concentrations, EPS isolated from a plasmodium showed higher radical scavenging ability than EPS isolated from the slime track material in both species. EPS from *Phy. oblonga* showed maximum DPPH scavenging activity (80.41%) at a concentration of 6 mg/ml, whereas that of ascorbic acid was 99.56%.

The EC50 is the concentration of antioxidant needed to obtain a 50% antioxidant effect, and is typically used as a parameter to express or compare the antioxidant capacity of different compounds. Lower EC50 values show a higher antioxidant activity [28]. EC50 values of the EPS samples and ascorbic acid are displayed in Table 6.

**Table 6:** EC50 values of the slime track and EPS samples from *Phy. oblonga* and *Ph. polycephalum*

Sample	EC50 value (mg/mL)
*Phy. oblonga* slime track	3.92 ± 0.22
*Phy. oblonga* EPS	3.59 ± 0.19
*Ph. polycephalum* slime track	5.99 ± 0.10
*Ph. polycephalum* EPS	4.87 ± 0.31
Ascorbic acid	0.78 ± 0.05

According to the EC50 data, the slime track and EPS samples from *Phy. oblonga* showed higher scavenging abilities than those from *Ph. polycephalum*. These data also indicated that EPSs and slime tracks from *Ph. polycephalum* and *Phy. oblonga* have comparable antioxidant capacity with some common edible mushrooms [29–32]. However, the antioxidant activities these samples were far smaller than ascorbic acid.

### In vitro cancer cell line cytotoxicity assays

**Fig. 4:**
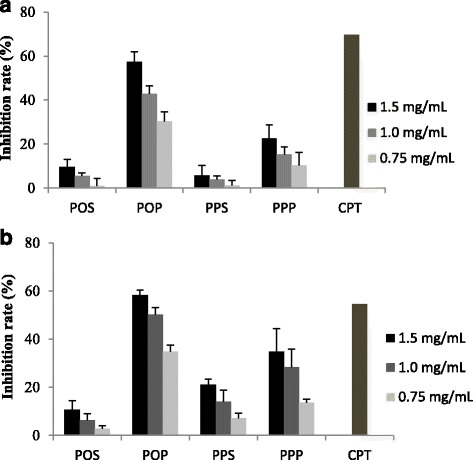
Growth inhibition of MCF-7 (**a**) and HepG2 (**b**) cancer cell lines by treating with crude EPS extracts from *Phy. oblonga* and *Ph. polycephalum* in comparison with camptothecin standard (CPT) with the concentration of 0.005 μg/mL via SRB assay. POS, POP, PPS and PPP were represented in the *Phy. oblonga* slime track, *Phy. oblonga* EPS, *Ph. polycephalum* slime track and *Ph. polycephalum* EPS

In this experiment, crude EPS and slime track samples from *Phy. oblonga* and *Ph. polycephalum* were subjected to in vitro cytotoxicity SRB assay with fibroblast and cancer cell lines. Cells were treated with EPSs ranging from 0.25 to 1.5 mg/mL and incubated for 48 h, and then the cell inhibitory rate was measured by using a spectrophotometer. The data obtained data are shown in Fig. 4.

The results indicate that EPSs possess different levels with respect to their toxicity effects against the cancer cell lines. At low concentrations (0.25–0.5 mg/mL), none of negatives effect on the proliferation of cancer cell lines were observed. However, EPSs were found to show anti-proliferation when the concentration increased from 0.75 to 1.5 mg/mL.

EPSs isolated from a plasmodium showed higher inhibition rates against the cancer cell lines than EPSs isolated from the slime tracks. Most notably, EPS from *Phy. oblonga* showed significantly higher inhibitory activities against MCF-7 and HepG2 when compared to that of *Ph. polycephalum.*

The half inhibitory concentrations (IC50) of the EPS sample from *Phy. oblonga* toward MCF-7 and HepG2 were found as 1.22 and 1.11 mg/mL, respectively. However, these activities are not comparable to the positive control (camptothecin).

Microbial EPS have been found to have anti-proliferation effects against HepG2 and MCF-7 cells. Wang et al. [9] reported that at the concentration of 600 μg/ml, purified EPS from *Lactobacillus plantarum* could suppress proliferation of HepG2 cells by 56.34% when treated for 72 h. In addition, Osama et al. [5] found that (IC50) of purified EPS from *Bacillus marinus* in the MCF-7 was 118.0 μg/mL after 48 h.

## Conclusions

Culturing myxomycete plasmodia is challenging, but it is possible with the right medium components (e.g., carbon source) selectively used for each species. For example, agar is more suitable for cultivation of *Phy. oblonga*, but glucose is a better carbon source for *Ph. polycephalum*.

The slime track and EPS samples from *Ph. polycephalum* and *Phy. oblonga* were found to consist of glucose, galactose and rhamnose. Among these, rhamnose was the major monomer of the EPS from *Phy. oblonga* and the slime tracks from both specie**s**, but EPS from *Ph. polycephalum* contained mainly glucose. This difference may be because of the use of different carbon sources or it could be simply just because of the unique nature of each species. However, since monomer composition is one of the major factors other than molecular weight, structure of the polymeric backbone and degree of branching which decide the biological activities of microbial EPSs. Thus, when one tries to enhance the amount of EPS production by altering medium composition and cultivation condition, the effect those conditions on EPS compositions and subsequently EPS activities should be taken in consideration along with the amount of EPS.

The slime tracks from both two species showed no antimicrobial activity, low anticancer activity, and moderate antioxidant activity. These results support the theory that function of the slime tracks of myxomycetes relates more to marking the area which has been exploited for food resources as the plasmodia migrate from one area to another.

On the other hand, EPS samples from the two species displayed significant inhibitory activities against *C. albicans* and *S. aureus*, and both of them had anticancer activities against MCF-7 and HepG2. More importantly, EPS from *Phy. oblonga* was found to have significantly higher inhibitory activities. The IC50 values of this sample on MCF-7 and HepG2 were 1.22 mg/mL and 1.11 mg/mL, respectively. The differences in biological activities of the slime track and the EPS which is still in contact with the plasmodia suggest that they probably have different functions for the particular species of myxomycetes. EPS purification should be considered in future works to enhance the biological activities.

Myxomycetes are a unique group of microorganisms which could be a potential source of bioactive compounds.

## Methods

### Materials

The strain of *Ph. polycephalum* used in the present study was obtained as a sclerotium from the Carolina Biological Supply Company (Burlington, North Carolina, USA). The *Phy. oblonga* plasmodium was generated from a fruiting bodies collected from a moist chamber culture prepared from forest floor litter.

Nutrient agar was used for the plasmodial culture of *Ph. polycephalum* (1.0 L of the nutrient agar contained 100 mL of a basal salt solution, 5.0 g of glucose, 2.5 g of yeast extract, 20.0 g of agar, and 900 mL of distilled water adjusted to pH 5.5). The basal salt solution contained 29.78 g of citric acid, 33.10 g of K_2_HPO_4_, 2.50 g of NaCl, 1.00 g of MgSO_4_.7H2O, 0.50 g of CaCl_2_.2H2O, and 1000 mL distilled water [23, 33].

Water agar was used for the *Ph. polycephalum* sclerotium and spore germination and plasmodial culture of *Phy. oblonga* (1.0 L of water agar consisted of 15 g of agar and 1000 mL of water).

Pathogenic microorganisms, including *Bacillus cereus* VTCCB 1005, *Escherichia coli* JM 109, *Samonella typhi* ATCC 19430, *Staphylococcus aureus* ATCC 43300 and *Candida albicans* ATCC 141 were used. The bacteria were grown on LB agar medium (1.0 L of LB agar containing 10.0 gof NaCl; 5.0 g of yeast extract; 10.0 g of peptone; 20.0 g of agar and 1000 mL of distilled water adjusted to pH 7.0) and the fungus was grown on Saboroud agar medium (1.0 L of Saboroud agar containing 40.0 g of glucose; 10.0 g of peptone; 20.0 g of agar and 1000 mL of distilled water adjusted to pH 5.5).

Cancer cell lines (breast carcinoma MCF-7 and liver carcinoma HepG2 cells) and fibroblast cells were grown in DMEM 10% FBS medium and mantained at 37 ° C in a 5% CO_2_ incubator.

### Plasmodial culture

Spore gernination, sclerotium activation and inoculum preparations of *Ph. polycephalum* and *Phy. oblonga* were carried out following Tran et al. [34, 35]. For plasmodial cultures and EPS production, a small piece of agar a containing actively growing plasmodium covered oatmeal flakes was transferred to a plate containing water agar (for *Phy. oblonga*) and nutrient agar (for *Ph. polycephalum*). The plasmodial cultures were incubated in the dark at 25 °C for 5 days, after which the slime tracks and plasmodia were collected.

### Isolation of slime tracks and EPSs from the plasmodial cultures

Slime tracks were simply scraped off the surfaces of the plasmodial cultures. For EPS isolation, fresh plasmodia were carefully collected in 10 mL of sterile distilled water without discrupting the plasmodium to avoid extracting the intracellular components. The sample was gentely vortexed and centrifuged at 9000 rpm, 4 °C for 25 min [5], the supernatant was transferred into another tube; chilled ethanol was added in which the ratio of ethanol to the sample was 3:1 (*v*/v). The tube was mixed well and set at 4 °C. The following day, the mixture was centrifuged at the conditions as described above, and the pellet was collected as EPS. Both EPS and slime track samples were dried at 60 °C, and this material served as dry crude EPS. The crude EPS was then dissolved in 10% (*w*/*v*) trichloroacetic acid to remove proteins [5]. The supernatant was precipitated with chilled ethanol and centrifuged at the conditions described above. The pellet, referred to as partially purified EPS, was dried at 60 °C and stored at 4 °C. Partially purified EPS was used for activity assessment and structural analysis.

### Chemical analysis

The total carbohydrate and protein content of the slime track and crude EPS samples were analyzed by using the phenol sulphuric acid method [36] and the Bradford method [37], respectively. The sulfate group content was analyzed with the barium chloride gelatin method [38].

### Monosaccharide composition analysis by TLC

Ten mg of partially purified EPSs was hydrolyzed in 1.0 mL 3 M triflouroacetic acid (100 °C, 8 h). After hydrolysis, TFA in the sample was removed by decompression evaporation. The hydrolyzed EPSs were re-dissolved in ultra-pure water. The supernatant was obtained by centrifugation at 13000 rpm for 20 min.

The hydrolyses were applied to silica gel plates using a developing solvent of butanol: acetone:pyridine: H_2_O [10:10:5:5 (*v*/v/v/v)]. Galactose, glucose and rhamnose were used as the standards. After TLC plate development, carbohydrate was visualized by spraying TLC plates with 1% aniline:1% diphenylamine:85% H_3_PO_4_ [5:5:1 (v/v/v)] and heating at 100 °C for 5 min to reveal the colored spots [39].

### Quantification of monomers by GC

Samples were prepared according to the Kakasy method [40] with some modifications. The hydrolyses were dissolved in pyridine containing 2.5% hydroxylamine hydrochloride; after inositol (as an internal reference) was added to the solution, it was allowed to react at 80 °C for 30 min and cooled down to room temperature. Hexamethyldisilazane (HMDS) and TFA were then added, the mixture was allowed to react for further 30 min at 45 °C and cooled down again. One mL of silylate derivative was subjected to a DB-1 column (30 m × 0.35 μm × 0.25 μm) of GC (Aligent 6890 N) fitted with a flame ionization detector (FID). The operating conditions were as follow: the N_2_ carrier gas rate was 1.0 mL/min; injection and detector temperatures were 280 °C and 300 °C, respectively; the column temperature was started at 60 °C for 1 min, then increased to 210 °C at the rate of 20 °C/min and maintained there for 5 min, and finally increased to 300 °C at the rate of 100 °C/min and maintained there for 10 min. Standard sugars (galactose, glucose, lactose, rhamnose and sucrose) with inositol as the internal standard were prepared and subjected to GC analysis separately in the same way.

### Antimicrobial activity

#### Well diffusion method

Antimicrobial activity of the EPS and slime track samples was determined using the agar well diffusion method [4]. A volume of 100 mL of the cell suspension of the pathogenic culture (10^8^ CFU/mL) was spread on the surface of a LB/Sabouraud dextrose agar plate using a sterile cotton swab. An amount of 100μL of the sample (5 mg/mL) was introduced into each well (8 mm in diameter) in the plate. The positive antibacterial control used was erythromycin (1.0 mg/mL) and the antifungal control was ketoconazole (1.0 mg/mL). Sterile distilled water was used as the negative control. The plates were incubated for 37 °C in 8 h. Antimicrobial activity was determined by measuring the diameter of the clear inhibition zone around each well.

#### Minimum inhibitory concentration (MIC)

MIC is the lowest concentration of an agent that inhibits the visible growth of a microorganism after overnight incubation [41]. MIC was carried out on the microorganisms that showed sensitivity to the samples and was done using the broth dilution method. MIC values were determined according Sen and Batra [42] with some modification. EPS/slime track samples were prepared with the concentration range of 0 to 20,480 μg/mL. One mL of sterile culture medium was placed in a sterile test tube containing 100 μl of microorganism suspension (10^5^ CFU/mL). Then, 1.0 ml of the EPS extract with a certain concentration was added to the mixture and incubated at 37 °C for 24 h. After that, turbidity of the mixture was measured by using a spectrophotometer at a wavelength of 600, OD_600_ value less than 0.01 was recorded as the MIC. Minimum bactericidal concentration (MBC) and minimum fungicidal concentration (MFC) were defined as the lowest concentration of extract which showed no evidence of microbial growth on nutrient agar plates. MBC and MFC were investigated to confirm the MIC results.

The radical scavenging activity of slime track/EPS samples was measured with the use of the DPPH assay described by Monaki et al. [43] with some modifications. In brief, 80 μL of the sample with a certain concentration ranging from 0 to 6.0 mg/mL was added to 120 μl of 0.02 mg/mL DPPH prepared in a methanol solution. The mixture was mixed gently and incubated at room teperature for 30 min in the dark. Then, the absorbance was measured at 517 nm and the inhibition was calculated using the following formula

$$ \mathrm{Scavenging}\  \mathrm{rate}\left(\%\right)=\left[\left({\mathrm{A}}_0\hbox{-} {\mathrm{A}}_1\right)/{\mathrm{A}}_0\right]\mathrm{x}100 $$

where A_1_ was absorbance of the sample and A_0_ was the absorbance of the control [44]. The antioxidant ability of the sample was expressed as an IC50 value, which was defined as the concentration of sample that inhibits the formation of the DPPH radical by 50%. An equal amount of methanol was added to the negative control, and ascorbic acid was used as the positive control.

#### Anticancer activity

The cytotoxicity of isolated EPS was determined using a sulforhodamine-B (SRB) assay [45]. The cancer cell lines (10^5^ cells/mL) were seeded in a 96-well microtiter plate and cultivated under standard conditions (5% CO_2_ at 37^°^ C). Stock solution of the EPS samples were prepared in distilled water and serially diluted with sterile medium to obtain the desired concentrations. One hundred μL of the sample was then added to each well and incubated for another 48 h for cell attachment. Cells were fixed by gently layering of cold 50% TCA and incubated at 4 °C for 1 h. The plate was then washed five times with distilled water and air-dried for 12 h at room temperature to avoid cell monolayer detachment. Cells were stained al least 15 min with 0.2% SRB dissolved in 1.0% acetic acid and subsequently washed 5 times with 1.0% acetic acid to remove unbound proteins. The plate was air-dried. A tris-base solution was added to the wells to solubilize the dye. The plates were shaken gently for 10 min on a mechanical shaker. Distilled water and camptothecin (0.01 μg/mL) were used as negative and positive controls, respectively. A blank contained culture medium without cells. The optical density (OD) of the plate wells was recorded using a microplate reader at 560 nm. Growth inhibition was calculated as.

$$ \%I=\left(1-\frac{\mathrm{A}}{\mathrm{B}}\right)\ x\ 100\% $$

where A and B represent the absorbance of the test sample and the control [45].

#### Statistical analysis

All experiments were done in triplicate and all data are expressed as mean ± standard eviation.

## Abbreviations

CFU: Colony forming unit
DPPH: 2,2-diphenyl-1-picrylhydrazyl
EPS: Exopolysaccharides
GC: Gas chromatography
IC50: Half inhibitory concentration
MBC: Minimum bactericidal concentration
MFC: Minimum fungicidal concentration
MIC: Minimum inhibitory concentration
TCA: Trichloroacetic acid
TFA: Triflouroacetic acid
TLC: Thin layer chromatography
